# High-Volume Center Experience with Laparoscopic Adrenalectomy over Two Decades

**DOI:** 10.3390/jcm11092335

**Published:** 2022-04-22

**Authors:** Milena Duralska, Jacek Dzwonkowski, Janusz Sierdziński, Sławomir Nazarewski

**Affiliations:** 1 Department of General, Vascular and Transplant Surgery, Medical University of Warsaw, 02-091 Warsaw, Poland; 19dzwonek@wp.pl (J.D.); slawomir.nazarewski@wum.edu.pl (S.N.); 2 Department of Medical Informatics and Telemedicine, Medical University of Warsaw, 02-091 Warsaw, Poland; jsierdzinski@wum.edu.pl

**Keywords:** laparoscopy, adrenalectomy, adrenal gland neoplasms, conversion to open surgery, postoperative complications, intraoperative complications

## Abstract

Background: Study aims to demonstrate single-institution two decades experience with lateral transperitoneal laparoscopic adrenalectomies. Methods: Retrospective study involved 991 operations grouped into 4 cohorts. Data was collected on the patients’ age, sex, side and size of the lesion, histopathological type, hormonal activity, conversion to open adrenalectomy, operating time, length of hospital stay, perioperative complications. Results: The operations were right-sided (*n* = 550), left-sided (*n* = 422), bilateral (*n* = 19). Mean tumor size was 41.9 mm. Histopathological examination revealed 442 adenomas, 191 nodular hyperplasias, 218 pheochromocytomas, 33 malignancies and 126 other lesions. 541 patients had hormonally active tumors. Mean operating time for unilateral laparoscopic adrenalectomy was 141 min. Mean length of hospital stay was 5.27 days. Intraoperative complications rate was 2.3%. Conversion rate was 1.5%. 54 of patients had 70 postoperative complications. Reoperation rate was 1%. Mortality rate was 0.1%. Statistically significant differences were found in all factors, apart from age, sex, side and size of the lesion, reoperations rate (*p* > 0.05). Conversions rate, complications rates, length of hospital stay were highest in the first group (*p* < 0.05). Operating time shortened in the first decade. Conclusions: Laparoscopic adrenalectomy is a safe procedure with negligible mortality. Conversions rate, perioperative complications rate, and length of hospital stay, significantly decreased over time.

## 1. Introduction

Although adrenal pathologies are rare, they are increasingly found incidentally as part of investigations for non-related reasons [1,2,3]. A variety of surgical approaches have been described to treat adrenal tumors, including open, endoscopic (laparoscopic, retroperitoneoscopic) and robotic methods [4,5]. The first laparoscopic adrenalectomy via a lateral transperitoneal approach was reported by Gagner et al. in 1992 [6]. Since then, the method became the standard approach in adrenal surgery due to its safety and feasibility [4,5].

Adrenal operations are uncommon in general surgical practice and the European Society of Endocrine Surgeons recommended that adrenalectomies should be performed in high-volume centers and established a minimum of 6 operations per year for a single center [4].

Contemporary evidence base on laparoscopic adrenalectomy consists mainly of reports on small series of patients. Larger datasets are distinctive for multi-institutional and national reports [7,8]. The largest single-institution case studies involve 400 to 650 patients [3,9,10,11,12,13]. Many factors affecting perioperative outcomes are discussed in the literature. Apart from surgeon experience, they include size of the tumour, histopathological type, hormonal activity, side of the lesion, sex of the patient [8,9,10,11,12,13].

The aim of this study is to demonstrate our single-institution, single-surgeon experience which spans over two decades and includes almost 1000 laparoscopic adrenalectomies performed via a lateral transperitoneal approach. We particularly focused to analyze patient outcomes with relation to evolving experience of the surgeon.

## 2. Materials and Methods

We retrospectively reviewed 991 laparoscopic adrenalectomies performed at a single academic medical center (Department of General, Vascular and Transplant Surgery, Medical University of Warsaw) between October 1997 to September 2017. Local Bioethics Committee approval for this research was obtained. All patients signed a written patients’ informed consent form for the operation.

Prior to surgery, all patients underwent endocrinological and anaesthesiological assessment. Pre-operative workup included hormonal evaluation and imaging (computed tomography, magnetic resonance imagery or positron emission tomography in selected cases), as well as pharmacological preparation when indicated. 

Indications to laparoscopic adrenalectomy included: (1) hormonally active tumor measuring <80 mm in diameter on imaging, (2) non-functioning benign tumor measuring 40–80 mm in diameter, (3) non-functioning benign tumor measuring <40 mm in diameter, if rapid growth, focal changes of density, irregular shape or high density on Computed Tomography scans were observed, (4) metastases measuring <80 mm in diameter. Patients with tumors measuring >80 mm in diameter (hormonally active and non-functioning tumors, as well as metastases), primary malignant tumors, or if reoperation was needed, were offered open adrenalectomy. Patient who had non-functioning benign tumors measuring <40 mm in diameter were observed.

All operations were performed by the same primary surgeon (Prof. Maciej Otto) and his team via the lateral transperitoneal approach. The surgical method has been described previously by our team [14,15]. Open adrenalectomies were not analyzed in this study. Neither retroperitoneoscopic approach nor robotic method were implemented in our center and thus are not included in this report.

In order to control for the operating surgeon’s increasing experience, the patients were grouped into 4 equal cohorts depending on when they underwent the procedure. Each cohort contained a consecutive number of patients with the first cohort representing the earliest cases and the last cohort representing the latest cases in the study period. The time periods for each group are defined in Table 1 and Table 2.

Data were collected on the patients’ age, sex, side and size of the lesion, histopathological type, hormonal activity, conversion to open adrenalectomy, operating time, length of hospital stay, intra- and postoperative complications, reoperations, and mortality. The tumor size was obtained from the histopathology report. Operating time was measured from skin incision to skin closure. The postoperative complications were categorized using the Clavien-Dindo classification system [16].

Patients who underwent bilateral and adrenal-sparing laparoscopic adrenalectomy were included in the study, although they were excluded from the analysis of operating time. Patients who required conversion to open adrenalectomy or additional elective procedure performed (e.g., hernia repair/cholecystectomy) were also excluded from the analysis of operating time.

### Statistical Analysis

The data were collected and stored using Microsoft Excel. Continuous variables were expressed as the mean, standard deviation (SD). Chi-square test and ANOVA were used to compare differences between the groups. Statistical significance was set at *p*-value < 0.05. Data were analyzed using STATISTICA 13.0 software.

## 3. Results

### 3.1. Patient and Tumor Characteristics

The study included a total of 991 laparoscopic adrenalectomies. Patient and tumor characteristics are summarized in Table 1. The mean (SD) age of the patients was 53 (14) years; *n* = 682 (68.8%) of the patients were female. With regards to the laterality, *n* = 550 had a right sided, and *n* = 422 had a left sided adrenalectomy. Bilateral adrenalectomy was performed in *n* = 19 patients. In 39 cases, an additional procedure was performed (cholecystectomy *n* = 29, hernia repair *n* = 10) concomitantly, during the same anesthetic time. The adrenal sparing procedure was performed in 21 patients. The mean (SD) size of the removed lesion was 41.9 (20) mm, range from 6 to 130 mm. Histopathological examination revealed 442 adenomas, 191 nodular hyperplasias, 218 pheochromocytomas, 33 malignancies and 126 other lesions. In all patients requiring bilateral adrenalectomies, tumors were histologically the same on both sides. Non-functioning lesions were identified in 450 patients, while 541 patients had hormones secreting lesions (*n* = 208 had catecholamine, *n* = 210 cortisol, *n* = 110 aldosterone, and *n* = 13 androgen).

### 3.2. Perioperative Data

Perioperative outcomes are outlined in Table 2. The mean (SD) operating time for unilateral laparoscopic adrenalectomy was 141 (47.1) min, range from 55 to 530 min. The mean (SD) length of hospital stay was 5.27 (4.68) days, range from 2 to 102 days. There were 23 (2.3%) intraoperative complications, with intraoperative bleeding being the most frequent one. Conversion to open adrenalectomy was performed in 15 (1.5%) patients. Indications for conversion are described in Table 3. Within the cohort, 54 (5.4%) patients experienced 70 postoperative complications. Postoperative blood transfusion was required in 41 patients (Clavien–Dindo = 2). Return to theatre was recorded in 10 patients, *n* = 7 cases had postoperative bleeding or hematoma, while *n* = 1 had a trocar-site herniation of the omentum, pancreatic fistula, and peritonitis each. Severe complications (Clavien–Dindo ≥ 3) were recorded in 1.6% of cases. Mortality was recorded in one patient (0.1%). A 73-year-old female with Cushing’s syndrome underwent right-sided laparoscopic adrenalectomy and was reoperated due to coagulopathy and bleeding (splenectomy was required). Postoperatively patient was hemodynamically unstable and required intensive care. Subsequently, another operation was carried out for peripancreatic fluid collection. Patient died due to multiorgan failure.

### 3.3. Evolution of Surgical Practice over Time

**Figure 1:**
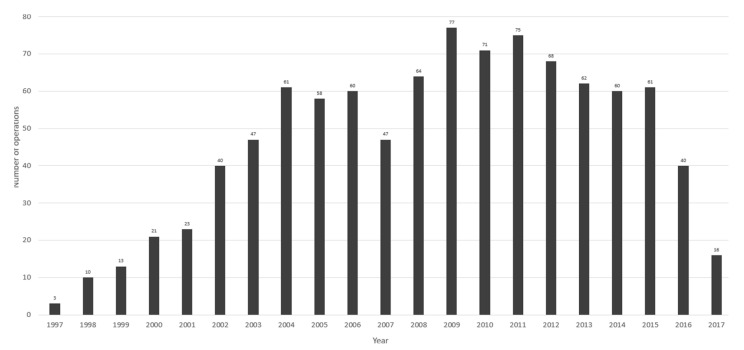
Annual number of operations.

In the first year of practice, 3 cases were recorded. The number of operations was gradually increasing over time, and since 2002, 40–77 laparoscopic adrenalectomies were performed each year. The annual number of operations is shown in Figure 1.

When comparing each cohort, there were no statistically significant differences between each group in terms of patient age, sex, side of lesion, size of lesion, reoperations rate (Table 1 and Table 2).

**Figure 2:**
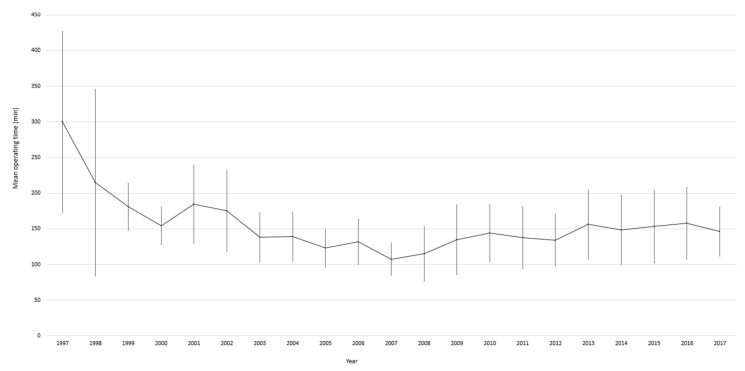
Annual mean operating time of unilateral laparoscopic adrenalectomy (excluded: bilateral adrenalectomies, conversion to open surgery, adrenal-sparing procedures, additional procedures performed).

**Figure 3:**
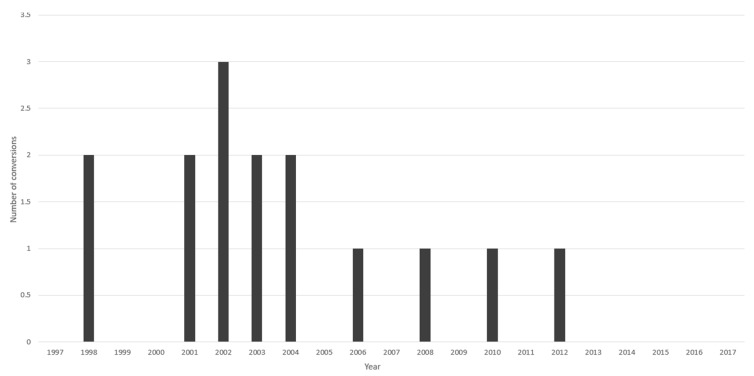
Annual number of conversions to open surgery.

The mean operating time of unilateral laparoscopic adrenalectomy was longest in group 1 (154 min) and shortest in group 2 (122 min). In group 3 and 4 the mean operating time was 138 min and 153 min, respectively (*p* < 0.0001) (Table 2). The operating time was found gradually shorter in the first decade (groups 1, 2 and partially group 3) and then reached a plateau in the second decade (Figure 2). Conversions rate, intraoperative and postoperative complications rates as well as length of hospital stay were highest in the first group and significantly decreased in groups 2–4 (Table 2). There were 11 conversions in the first group, and reduced to two conversions in the second group and to one conversions in the subsequent groups (*p* = 0.0002). In addition, based on a view of annual performance, there is a reduction in conversion rate over time (Figure 3). Intraoperative complications developed in 11 cases in the first group and reduced to 4 cases in groups 2–4 (*p* = 0.002). The number of postoperative complications in groups 1–4 was 47, 5, 5, 13, respectively (*p* < 0.0001). Mean length of hospital stay decreased from 7 days in the first group to 4.8 and 4.6 days in groups 2–4 (*p* < 0.0001). The number of patients who underwent an adrenal sparing procedures significantly increased over time from one procedure in the first group to 9 procedures in the last group (*p* = 0.034) (Table 1).

## 4. Discussion

This study presents an analysis of almost 1000 laparoscopic adrenalectomies via lateral transperitoneal approach performed by a single surgeon over the 20 years. This is the largest published series describing experience with laparoscopic adrenalectomy in a single institution. Our study demonstrates an overall low rate of perioperative complications and a negligible mortality rate following laparoscopic adrenalectomy. Moreover, complications rate was demonstrated to reduce gradually over time and operating time was gradually reduced in the first decade of practice before reaching a plateau. The length of hospital stays also significantly decreased over time.

As a high-volume, national referral center, our institution performs about 50 laparoscopic adrenalectomies per year. It is in line with the consensus statement of the European Society of Endocrine Surgeons, stating that adrenal surgery should be performed in departments with at least 6 adrenalectomies per year, and at least 12 adrenalectomies per year—for management of adrenocortical cancer [4].

When comparing groups with regards to the tumor characteristics, we found statistically significant differences in histopathological type and hormonal activity (Table 1). The differences result from a small number of patients with malignant lesions and androgen-secreting tumors compared to other types, respectively.

There is controversy regarding the safety and feasibility of laparoscopic adrenalectomy as management for malignant lesions. The European guidelines recommend the open method as a standard technique [5,17]. In our center, suspected primarily malignant lesions as well as metastatic lesions measuring >80 mm in diameter, in preoperative assessment are managed with an open operation. In our series, the number of lesions found to be malignant during histopathological examination decreased over the study period, likely due to improvements in preoperative diagnostic tools [3,18]. Pędziwiatr et al., based on experience with 500 laparoscopic adrenalectomies, reported that the laparoscopic method can be a good alternative to open resection for the treatment of malignant adrenal tumors, when performed by an experienced team [10].

The mean operating time of unilateral laparoscopic adrenalectomy in our series was 141 min and decreased with increasing surgeon experience. In the literature, the mean operating time is reported to be between 83 and 240 min [3,7,8,9,10,11,12,13,19,20,21,22,23,24,25]. Apart from the operating surgeons’ experience, other factors may affect operating time and include: patient sex, obesity, tumor size, and presence of malignancy [10,19,25].

The overall rate of conversion to open surgery in our series was 1.5% and was significantly decreased over time. Other authors reported conversion rate ranging from 0.8% to 6.2%, although in majority of publications the conversion rate was more than 3.5% [3,8,9,10,12,13,19,20,21,22,25]. In our study, the most common indication for conversion to open surgery was suspicion of incomplete tumor resection and suspicion of malignancy (e.g., inferior vena cava infiltration). In a group of patients requiring conversion, the most common pathology was pheochromocytoma (60%) followed by malignancy (20%). Size could also be an important risk factor for conversion to open adrenalectomy, as mean size of the tumor requiring conversion in our series was 52 mm, whereas overall mean size of the removed tumor was 41.9 mm. Right sided tumors constituted 73% of all tumors requiring conversion to open adrenalectomy in our series. Factors defined as increasing risk for conversion vary in the literature, and involve pheochromocytoma, tumor size measuring > 50–80 mm, Body Mass Index > 24 kg/m^2^ [12,22,25,26].

Many authors have used Clavien–Dindo classification to describe the complication rates of laparoscopic adrenalectomy. The average rate of perioperative complications of laparoscopic adrenalectomy reported in the literature ranges from 0 to 15.5%, with bleeding being the most frequent one [3,7,8,9,10,11,12,13,20,21,22,23,25]. Postoperative complications occurred in 5.4% of patients in our study. The complications rate was found to reduce gradually over time, concomitant to the operating surgeon’s increasing experience. Within our patients, complications occur most frequently in patients who had pheochromocytoma and cortisol—secreting tumors. Battistella et al., in their 35 years of experience with 520 adrenalectomies reported zero peri-operative complications at 30-days and concluded that patients requiring adrenalectomy should be treated only in experienced centers [3]. Other authors report that factors associated with development of perioperative complications include: tumor size > 60 mm, American Society of Anesthesiology (ASA) class 3 or 4, presence of pheochromocytoma or cortisol-secreting adenoma, conversion to open/hand-assisted operation, and left-sided tumors [12,13,25].

The overall mean length of hospital stay in our series was 5.27 days. When comparing the groups with regards to when they were performed, the length of stay gradually reduced from 7 days in the earliest cohort to 4.6 days in the most recent cohort. In the literature, mean length of hospital stays range between 1 and 12 days [3,7,8,9,10,11,12,13,14,20,21,22,23,25]. Factors associated with prolonged length of stay, discussed in the literature, include presence of postoperative complications, day of the week of operation (Thursday or Friday), use of drainage, pheochromocytoma, specimen size > 90 mm, age > 65 years, bilateral adrenalectomy, (ASA) class 3 or 4 [13,25,27,28].

The mortality rate in our series was 0.1%—this is comparable to other centers which quote a mortality ranging from 0% to 0.8%, emphasizing the overall safety of the laparoscopic adrenalectomy [9,10,13,22,23].

Limitations of this study are mostly related to its observational retrospective nature, as well as all operations being performed by the single surgeon and his team. Our Department is a national referral center. Postoperatively, many patients were followed up in their local hospitals, so our long term follow up data is partially incomplete and long-term outcomes were not analyzed. Another limitation is lack of data about the type of surgical equipment used during each operation. Development of technology over the two decades of practice could influence the outcomes. Moreover, information about the exact volume of intraoperative blood loss, Body Mass Index were not documented in available operating records.

## 5. Conclusions

This large single-center case series adds to the existing body of evidence on laparoscopic approach to adrenalectomy. Our data supports the safety and feasibility of the procedure and extends the evidence that laparoscopic adrenalectomy is a safe procedure with negligible mortality. Conversion and perioperative complication rate as well as length of hospital stay significantly decrease over time with increasing surgeon and institutional experience and excellent cooperation with endocrine and anesthesiology teams in perioperative care of patients undergoing laparoscopic adrenalectomy. We think that some lengthening of the operation time is acceptable if performed in order to achieve higher quality of surgery, reduce complications rate, maintain oncological margins and avoid conversion. Based on our material where all operations were performed by the same primary surgeon and his team, we can also conclude that gaining mastery in adrenalectomy can be very beneficial to the patients as above can assure very good outcomes.

## Figures and Tables

**Table 1:** Patient and tumor characteristics.

	Overall	Group 1	Group 2	Group 3	Group 4	*p*-Value/Test
**Number of operations**	991	247	248	248	248	
**Time period**	October 1997–September 2017	October 1997–May 2005	May 2005–June 2009	June 2009–November 2012	Novmber 2012–September 2017	
**Mean age (years)**	53	52	53	54	54	*p* = 0.051/2.2 **
**Sex:**						*p* = 0.707/1.4 *
- Female	682	174	175	165	168	
- Male	309	73	73	83	80	
**Side:**						*p* = 0.123/10.03 *
- Right	550	148	147	127	128	
- Left	422	92	98	118	114	
- Bilateral	19	7	3	3	6	
**Adrenal-sparing procedure**	21	1	3	8	9	*p* = 0.034/8.68 *
**Mean size of the lesion (mm)**	41.9	42.2	39.9	44.4	41.1	*p* = 0.144/1.81 **
**Histopathology (number of tumors):**						*p* < 0.0001/24.72 *
- Pheochromocytoma	218	46	60	48	64	
- Adenoma	442	131	104	108	99	
- Nodular Hyperplasia	191	34	61	48	48	
- Malignancy	33	15	7	8	3	
- Other	126	28	19	39	40	
**Hormonal activity:**						*p* < 0.0001/41.2 *
- Nonfunctioning	450	116	111	116	107	
- Aldosterone-secreting tumor	110	37	36	21	16	
- Glucocorticosteroid-secreting tumor	210	50	42	63	55	
- Catecholamine-secreting tumor	208	43	59	46	60	
- Androgen-secreting tumor	13	1	0	2	10	

* Chi-square; ** ANOVA.

**Table 2:** Perioperative outcomes.

	Overall	Group 1	Group 2	Group 3	Group 4	*p*-Value/Test
**Number of operations**	991	247	248	248	248	
**Time period**	October 1997–Septmber 2017	October 1997–May 2005	May 2005–June 2009	June 2009–November 2012	November 2012–September 2017	
**Operating time of unilateral laparoscopic adrenalectomy (mean, min)**	141	154	122	138	153	*p* < 0.0001/24.67 **
**Length of hospital stay (mean, days)**	5.27	7	4.8	4.6	4.6	*p* < 0.0001/15.49 **
**Intraoperative complications:**	23	11	4	4	4	*p* = 0.002/30.54 *
- Bleeding	13	10	1	0	2	
- Spleen injury	8	0	3	4	1	
- Liver injury	1	1	0	0	0	
- Bowel injury	1	0	0	0	1	
**Conversion**	15	11	2	1	1	*p* = 0.0002/19.25 *
**Postoperative complications:**	70	47	5	5	13	*p* < 0.0001/30.44 *
Clavien-Dindo grade I						
- Port-site hematoma	8	7	0	0	1	
- Hematoma in adrenal loggia	2	1	1	0	0	
- Hypokalemia	2	1	0	0	1	
Clavien-Dindo grade II						
- Clostridium difficile infection	1	1	0	0	0	
- Blood transfusion	41	27	3	4	7	
Clavien-Dindo grade IIIb						
- Bleeding	7	4	1	0	2	
- Trocar-site herniation	1	0	0	0	1	
- Pancreatic fistula	1	1	0	0	0	
- Peritonitis	1	0	0	1	0	
Clavien-Dindo grade IVa						
- Hemorrhagic pancreatic necrosis	1	1	0	0	0	
- Cardiac insufficiency	4	3	0	0	1	
Clavien-Dindo grade V						
- Death	1	1	0	0	0	
**Reoperation**	10	5	1	1	3	*p* = 0.215/4.47 *

* Chi-square; ** ANOVA.

**Table 3:** Indications for conversion to open surgery.

	Operation Number	Side	Size (mm)	Histopathology	Reason for Conversion
1	6	Right	50	Pheochromocytoma	Adhesions
2	14	Right	30	Pheochromocytoma	Adhesions, enlarged liver
3	54	Right	55	Adenoma (inactive)	Suspicion of incomplete tumor resection Tumor totally located behind inferior vena cava
4	62	Bilateral	25/25	Pheochromocytoma	Left side: Bleeding (splenic artery)
5	73	Right	40	Adenoma (Cushing’s syndrome)	Megacolon
6	80	Right	90	Adrenocortical carcinoma (Cushing’s syndrome)	Suspicion of malignancy, Large tumor
7	97	Right	50	Pheochromocytoma	Suspicion of incomplete tumor resection
8	113	Right	45	Pheochromocytoma	Suspicion of incomplete tumor resection
9	138	Right	60	Pheochromocytoma	Tumor totally located behind inferior vena cava
10	188	Right	50	Adenoma (Conn syndrome)	Adhesions
11	220	Left	65	Metastasis	Inferior vena cava infiltration
12	334	Right	70	Metastasis	Inferior vena cava infiltration
13	448	Right	75	Pheochromocytoma	Tumor totally located behind inferior vena cava
14	558	Left	50	Pheochromocytoma	Severe kyphoscoliosis, anatomical topography abnormalities
15	749	Bilateral	20/20	Pheochromocytoma	Left side: Adhesions, megacolon, enlarged spleen

## Data Availability

Not applicable.
